# Diagnosis and Treatment of Lower Motor Neuron Disease in Australian Dogs and Cats

**DOI:** 10.1155/2018/1018230

**Published:** 2018-08-06

**Authors:** A. M. Herndon, A. T. Thompson, C. Mack

**Affiliations:** ^1^University of Queensland School of Veterinary Science, Gatton, QLD 4350, Australia; ^2^Veterinary Specialist Service, Underwood, QLD 4127, Australia

## Abstract

Diseases presenting with lower motor neuron (LMN) signs are frequently seen in small animal veterinary practice in Australia. In addition to the most common causes of LMN disease seen world-wide, such as idiopathic polyradiculoneuritis and myasthenia gravis, there are several conditions presenting with LMN signs that are peculiar to the continent of Australia. These include snake envenomation by tiger (*Notechis *spp.), brown (*Pseudonaja *spp.), and black snakes (*Pseudechis *spp.), tick paralysis associated with* Ixodes holocyclus* and* Ixodes coronatus*, and tetrodotoxins from marine animals such as puffer fish (Tetraodontidae spp.) and blue-ring octopus (*Hapalochlaena *spp.). The wide range of differential diagnoses along with the number of etiological-specific treatments (e.g., antivenin, acetylcholinesterase inhibitors) and highly variable prognoses underscores the importance of a complete physical exam and comprehensive history to aid in rapid and accurate diagnosis of LMN disease in Australian dogs and cats. The purpose of this review is to discuss diagnosis and treatment of LMN diseases seen in dogs and cats in Australia.

## 1. Introduction, History, and Physical Examination

Lower motor neuron disease (LMND) broadly refers to conditions that preferentially affect the motor nerve bodies originating in the ventral horn of the spinal cord grey matter, their axons, the neuromuscular junction, and the muscle fibre. Disruption of motor unit function results in diminished motor function (e.g., paresis or paralysis) of the affected region, flaccid muscle tone, and diminished or absent reflex arcs. Depending on the region and type of disease, this change in function may be regional, such as megaoesophagus seen in focal myasthenia gravis, or generalised, such as tetraparesis with pharyngeal and respiratory muscle paralysis that can be seen with tick paralysis cases. Lower motor neuron disease is the result of a wide variety of underlying disease pathologies. Immune-mediated targeting of nerves or motor endplate is seen in myasthenia gravis and idiopathic polyradiculoneuritis [1–3].

The importance of a good history cannot be overemphasized. As certain causes of LMND in Australia show a strong regional distribution (e.g., tick paralysis), understanding the lifestyle and recent travel history of the pet may assist in shortening the list of differential diagnoses [4–9]. In addition to travel history, other important questions include the following: recent history of antigenic stimulation (e.g., vaccine); use of acaricides; onset, duration, and progression of clinical signs; sighting of ticks or snakes in the pet's environment; history of similar, previous events; and history of raw or undercooked animal products in the diet.

Neurolocalisation is a critical first step in identifying a disease with a LMND component. A summary of clinical abnormalities on neurologic exam and their relationship to LMND is included in Table 1. The clinical findings of a good neurologic exam are frequently enough to localise the neurologic lesion to brain, brain stem, spinal cord segments, or lower motor neuron. The common theme among all neurologic abnormalities is the failure of the motor unit to function normally, despite normal sensory input.

The clinical hallmark of lower motor neuron disease is skeletal muscle weakness, although there are examples of smooth and cardiac muscle involvement associated with diseases affecting the lower motor neuron [10]. LMND weakness is infrequently global, or involving all muscle groups equally. In most instances there will be a progression of signs with the pelvic limbs being affected first followed by thoracic limbs, oesophagus, and then cranial motor nerves to the face, pharynx, and larynx [3, 11–15]. Occasionally, disease is limited to a specific region, such as oesophagus in myasthenia gravis [3, 11, 13, 16]. Even less commonly, LMND signs may first be seen in the thoracic limbs [11]. Gait abnormalities such as goose-stepping, crossing limbs, spasmodic movements, or head tilts and turns are not consistent with LMND and disorders of the CNS should be considered.

It is important to keep in mind that in LMND the sensory component of the nervous system is intact. Therefore, patients with LMND have intact nociception, proprioception, and spatial awareness. The skeletal muscle weakness may have the appearance of ataxia, but with support these patients will attempt normal responses to postural reactions and positioning. Postural reaction and nociceptive testing are important tools for differentiating LMND from spinal or brain disease. Polyneuropathies affecting both motor and sensory pathways may present with ataxia, and this may be seen along with classical LMN signs. Another important discriminator is that alterations in mentation and/or seizures are always associated with forebrain disease and are not consistent with LMND as a sole aetiology.

Assessment of ventilation parameters can be critical in advanced or rapidly progressive LMND. Patients may be tachypneic, but due to weakened diaphragm and intercostal muscles the patient may be seriously underventilated. Dogs with tick paralysis often demonstrate a classic expiratory “grunt.” In the absence of severe pulmonary disease, identifying hypercapnia on venous blood gas sample is highly suggestive of hypoventilation and is an indication for ventilatory support. Lower motor neuron disease patients unable to maintain adequate ventilation require rapid intervention, possibly intubation with intermittent positive pressure ventilation or maintenance via mechanical ventilator.

Dysphonia, dysphagia, and megaoesophagus are characteristics of some LMN diseases, especially with tick paralysis and myasthenia gravis [15, 17–21]. Ptyalism, slow or absent gag, slow or absent swallow on laryngeal palpation, and regurgitation are all consistent with pharyngeal, laryngeal, and oesophageal disease. As normal laryngeal function is required for the reflex movement of the epiglottis to protect the airway, patients with abnormal laryngeal function or those with passive regurgitation are at extremely high risk of aspiration. It is important that any patient with evidence of dysphagia or megaoesophagus be maintained in sternal recumbency with the head elevated at all time. As many LMND patients are unable to support their heads, stacks of towels or pillows may be required to keep the patient in an upright position.

Nutritional requirements of dogs and cats with LMND will vary depending on the severity of disease and the effect on the animal's ability to prehend food. In cases of focal or pelvic limb weakness, appetite, drinking, and eliminations are frequently normal. However, patients with laryngeal paralysis or megaoesophagus may even benefit from a gastric or nasogastric feeding tube in order to provide nutrition in the face of an inability to prehend food. Any patient that is unable to eat within three days or where the prognosis is such that they are unlikely to be able to safely prehend food within the next several days or weeks is a candidate for a supportive enteral feeding catheter, for example, a percutaneous gastric feeding tube.

The degree and duration of supportive care required for animals affected by LMN diseases completely depend on the severity of disease and expected duration of clinical signs. Specific diseases are discussed in the next section. It follows logic that the more support a patient initially requires in hospital, the more prolonged their recovery may be. Cases of polyradiculoneuritis, for instance, may require days to weeks in hospital followed by weeks or even months of home care to fully resolve [11, 22, 23]. Some cases of tick paralysis respond rapidly to tick removal and/or antiserum and may improve from nearly lateral recumbency to normal ambulation within 24 to 48 hours [19]. The prognosis associated with the various LMND is discussed in the next section.

## 2. Idiopathic Polyradiculoneuritis

Idiopathic polyradiculoneuritis, or acute canine polyradiculoneuritis (ACP), is an ascending lower motor neuron paralysis first identified in dogs in the southern United States after exposures to raccoon saliva by way of a bite wound [24–26]. Identical signs were described in dogs not exposed to raccoon bites and in these dogs the condition eventually became known as idiopathic polyradiculoneuritis [11]. However, the original moniker of “Coonhound paralysis” still persists even though that only describes a specific subset of cases.

Polyradiculoneuritis is an immune-mediated disease that can be triggered by a wide variety of stimuli, including vaccines, raccoon saliva, and some infectious agents (including* Toxoplasma gondii*) [25, 27–30]. The precise pathophysiology is not known for all cases of ACP, but immune targeting of gangliosides in nerve bodies and axons has been identified. Anti-ganglioside autoantibodies are also seen in the human disease Guillian-Barré, and canine polyradiculoneuritis is seen as a homolog to Guillian-Barré in people [31]. There is no age, breed, or sex predilection associated with development of ACP.

Diagnosis of ACP is made based on presenting clinical signs and ruling out other cases of LMND. A complete history and thorough tick-check are a necessary first step in ruling out other common causes of LMND such as snake bite, tick paralysis, or myasthenia. A Snake Venom Detection Kit may be necessary to help rule out snake envenomation if there is a high enough index of suspicion. Acetylcholine receptor antibody titres are valuable to attempt to rule out acquired myasthenia gravis.* Toxoplasma gondii* serology should be considered in all cases of ACP diagnosed in Australia [29].

Clinical signs of ACP typically begin in the pelvic limbs and slowly (over days) progress to involve the thoracic limbs and cervical muscles. In severe cases, motor function to the larynx and facial nerves is affected and dysphonia and absent swallow are possible [22, 23, 28, 32–34]. Paralysis of respiratory muscles is not common but possible. Motor function to the perineum and tail are typically spared and ACP dogs are able to wag their tail and perform normal, intentional urination and defecation [11]. ACP patients are typically bright and alert. Megaoesophagus is not a characteristic of ACP in dogs. Hence, a lack of a megaoesophagus may support a diagnosis of ACP.

Although ACP is immune-mediated, immunosuppression is not advised. There have been no controlled trials investigating this in veterinary patients, and this advice is based on strong evidence in the human literature against employing corticosteroids in the treatment of Guillian-Barré [35]. The use of human intravenous immunoglobulin (IVIG) has been investigated in one study, but the benefits of immunoglobulin therapy were not clear [22].

The mainstay of treatment of ACP is supportive care including nutritional support and physical therapy. ACP patients have normal sensory input and attention should be paid to provide adequately soft bedding and regular repositioning to avoid sores and discomfort. Every effort should be made to maintain sternal recumbency to facilitate easier eating and interaction with their environment. It is important to manage owner expectations as well in cases of ACP. Complete recovery is common (barring significant complication such as aspiration pneumonia) but can be prolonged. Most patients will gradually recover within a few weeks, but complete recovery may require several months of supportive nursing care [11, 23–25, 27, 28, 33].

## 3. Myasthenia Gravis

Myasthenia gravis (MG) is the result of immune-mediated targeting of the sarcolemmal acetylcholine receptors in the neuromuscular junction. The resulting blockade and associated remodelling cause insensitivity to acetylcholine and a failure of excitatory signalling to propagate to the myocyte and a flaccid paresis/paralysis [3].

Myasthenia gravis can be seen at any age, but age at onset of clinical signs is distributed largely along two peaks, with one seen in young adulthood between two and four years of age, and a second one later in life between the ages of nine and thirteen [18, 21]. In dogs, MG is associated with a mediastinal mass approximately 3.4% of cases, whereas in cats the proportion is markedly higher with around half (52%) of all MG cats in one case series having a concurrent diagnosis of a mediastinal mass [36, 37].

There are known breed predispositions for MG in both dogs and cats. Somali and Abyssinian breeds appear to be overrepresented among cats and the Akita, German Shepherd dog, German Shorthaired Pointer, Newfoundland, and most terrier breeds are overrepresented among dogs [36–39].

Presenting clinical signs can vary from mild to severe, focal or generalised, and include weakness or paresis, collapse, megaoesophagus, and dysphonia. Signs are commonly more noticeable in the pelvic limbs. Owners typically report exercise intolerance deteriorating into a short, stilted gait which resolves after rest being a frequent complaint. In one study, approximately 43% of myasthenic patients did not have clinically detectable limb weakness at the time of diagnosis [37]. Some dogs and cats will present with only megaoesophagus or dysphonia and no evidence of limb weakness [16, 20, 21, 39].

Myasthenia gravis has been reported as a paraneoplastic syndrome associated with numerous tumours. Most commonly associated with MG are mediastinal masses, especially thymoma, as mentioned above. However, various other sarcomas and haematopoetic tumours have also been associated with the onset of MG [40–43].

A definitive diagnosis of MG requires demonstration of a positive antibody titre of greater than 0.6 nmol/L in dogs and 0.3 nmol/L in cats to acetylcholine receptors [20]. Occasionally, patients presenting early in the course of disease may have titres within the reference interval [44]. These patients may develop a positive titre if rechecked in 2-3 weeks. A very small percentage of tetraparetic or fulminant MG patients may have a negative titre, but this is reported as less than 2% [20, 45]. At the time of this publication, the only location running ACh receptor antibody titre is in North America and the turn-around time for testing a sample from an Australian patient is close to three weeks. Therefore, a presumptive diagnosis is usually required while awaiting the definitive diagnosis. Because of the variety of clinical presentations for acute MG it can be difficult to differentiate acute, fulminant MG from other causes of acute LMND based on presenting signs alone. For instance, as many as 10% of acute MG patients may not present with megaoesophagus. In such a case, fulminant MG may be difficult or impossible to differentiate from ACP.

In cases where snake envenomation and tick are less likely (or have been ruled out) and the index of suspicion for MG is high, the clinical response to a test dose of edrophonium or pyridostigmine may be a good diagnostic option. Edrophonium has been unavailable on the Australian market for some time. However, if available, it provides an immediate, but very short lived, reversal of clinical signs in most MG cases of mild to moderate severity. Edrophonium is dosed at 0.11-0.22 mg/kg IV once for dogs and 0.25-0.5 mg per cat. The effect is typically seen within seconds and lasts less than two minutes. Alternatively, neostigmine bromide can be administered at a dose of 0.02 mg/kg given slowly IV. The effects of a single dose of neostigmine are not as dramatic as those seen with edrophonium, but a good clinical response (increased strength, ability to walk) within 15 to 30 minutes would support a diagnosis of MG. Both drugs must be used with caution in cats as they are more sensitive to the cholinergic side-effects than dogs. Pretreatment with atropine is recommended in all cats and should be kept on-hand in the event of an adverse reaction in dogs shortly after administration. It is important to remember that a positive response to acetylcholine esterase inhibitor is not pathognomonic for MG as other diseases of the neuromuscular junction may also experience a transient response.

Treatment of MG in dogs and cats focuses on increasing the amount of acetylcholine available at the neuromuscular junction by inhibition of acetylcholinesterase enzyme. Pyridostigmine bromide (Mestinon®) is dosed at 1-3 mg/kg by mouth every eight to twelve hours to start and the dose may be slowly increased over a period of weeks if necessary to see clinical improvement. Side-effects (nausea, diarrhoea, salivation, and lacrimation) are usually mild and resolve with time but are occasionally significant in some patients and require dose adjustments or concurrent administration of atropine to minimise side-effects. Nutritional support may be a challenge in MG patients with concurrent megaoesophagus. Elevated feeding, slurry feeding, and even use of gastric feeding tubes may be necessary to aid in passive movement of food into the stomach and to limit the possibility of an aspiration event.

Immunosuppression is not routinely necessary in mild or focal cases of MG and the myopathy associated weakness seen with high-dose steroid administration may only complicate the disease [46]. However, in severe or refractory cases of MG, immunomodulatory therapy may need to be considered. Corticosteroids such as prednisolone dosed at 0.5 mg/kg/day in dogs or cats is usually appropriate to start [20]. The dose is conservative at first to avoid side-effects such as muscle weakness but can be gradually increased over a period of days and weeks if needed to immunosuppressive dosing of around 1.5 mg/kg/day. If enteral administration of medication is complicated by megaoesophagus, parenteral dexamethasone at 0.15 mg/kg/day may also be considered. Cyclosporine or mycophenolate mofetil may be an alternative in patients where steroid use is contraindicated (diabetic, septic/pneumonia cases) [46, 47]. Azathioprine can be an excellent adjunct therapy or even a good long-term monotherapy, but slower onset of action may limit its utility in acute or fulminant cases of MG [20, 48]. Additionally, azathioprine is not appropriate for use in cats with MG as it is associated with neuromuscular blockade in this species. In human cases of MG, plasmapheresis and intravenous immunoglobulin treatments are associated with clinical improvement, but these therapies are not available, practical, or appropriate for most canine or feline patients [49–52].

Spontaneous remission of disease is possible in cases of MG in dogs. In fact, some reports suggest nearly 89% of dogs may experience spontaneous remission [53]. Remission does not appear to be a characteristic of the disease in cats [36]. Clinical remission of MG in dogs may occur within as little as one month but on average occurs around six months after diagnosis [53].

## 4. Tick Paralysis

Tick paralysis is not exclusive to Australia. The disease is seen very infrequently in the Pacific Northwest and Atlantic South East of the United states and rarely in Europe [15, 54, 55]. By comparison, tick paralysis is fairly common in Australia with one study describing over 3,400 cases in eastern Australia over just two tick seasons, from September 2010 to January 2012 [56]. The disease is associated with toxins produced by the salivary glands of hard-bodied ticks in the genus* Ixodes*, specifically* Ixodes holocyclus, coronatus*, and* neumann* [4, 7, 15, 57–59]. This is in contrast to tick paralysis in the United States that is associated with the genus* Dermacentor* [54, 55]. The exact mechanism of holocylotoxin-induced LMND is incompletely understood. The effect of the toxin appears to be focused on the presynaptic surface of the motor endplate and appears to block calcium influx, thereby preventing depolarization of nerve endings and propagation of signal across the neuromuscular junction [12, 15].

Cases of tick paralysis show a very strong geographical and seasonal distribution. The* Ixodes* ticks are distributed along the east and Southeastern coasts of Queensland, New South Wales, and Victoria, and roughly follow the native habitat of their preferred bandicoot and possum hosts [7, 15, 17, 60–63]. Only the female tick has long enough mouthparts to pierce and hold fast. Therefore, only females feed for a long enough time to produce tick paralysis. This helps explain much of the seasonality of tick paralysis with the numbers of female ticks seeking large meals in preparation for egg laying peak in the spring and early summer months. Tick paralysis cases can be seen year-round, but nearly three-quarters of all cases occur between September and December and a further 14% of cases over the summer months [17, 56].

Tick feeding behaviour is not a simple “attach and start feeding” process. Over the first few days the tick will draw blood in and out of her mouthparts as she prepares both herself and the host's local environment for a larger blood meal. Over these several days, the amount of holocylotoxin the salivary glands produce increases [15]. For this reason, ticks typically must remain attached for several days to see disease, with paralysis starting on the third day of feeding and progressing on subsequent days. This also highlights the importance of daily tick-checks and rapid-kill acaricides as effective preventative measures as they prevent tick attachment long enough to allow clinical disease to manifest. There are currently several acaricides on the market in Australia with documented rapid kill of* Ixodes* species [64–67]. Although no definitive studies have been published to date, early evidence supports a possible effect of isoxazoline parasiticides in decreasing the incidence of tick paralysis in Australia [68].

Definitive diagnosis of tick paralysis is almost always made based on finding an engorged tick or a recent “crater” or site where the tick was recently attached. When tick paralysis is a likely differential, it is advised to clip all hair on the body and perform a thorough search for embedded ticks. Particular attention must be paid to the head and neck, thorax, areas of skin folds (axilla, vulva, and groin), and the interdigital spaces [63]. Successful treatment and a positive outcome are impossible unless all ticks attached to the patient are identified and removed. It is not necessary to find an engorged tick as the offending tick may be early in her feeding or have finished her feed and detached prior to the onset of significant clinical signs. Finding any tick or “crater” from attachment is diagnostic. It is common practice to apply a topical acaricide, such as permethrin or fipronil, to kill any ticks that may have been missed during the search. These treatments do not substitute for a comprehensive search and tick removal as killing an already attached and feeding tick is not as effective at resolving clinical signs as mechanically removing the feeding tick.

The clinical signs of tick paralysis commonly start as an ascending limb paralysis first noted in the pelvic limbs and eventually involving the thoracic limbs. Involvement of the larynx and oesophagus as well as paralysis of the facial nerve is possible as the disease progresses [11, 15, 56, 63]. Advanced cases, particularly in cats, may see dilated and unresponsive pupils as the oculomotor nerve is involved [62]. Asymmetry in presenting signs is occasionally encountered with thoracic limbs more affected than pelvic limbs or right and left cranial nerves asymmetrically affected [14]. A clinical scoring system for tick paralysis is commonly used to standardise the assessment of tick paralysis patients [17] (Table 2). This scoring system considers the motor function (mild paresis through to lateral recumbency, graded 1-4) as well as respiratory score (no respiratory problems through to severe distress and cyanosis, graded A-D). Use of this scoring terminology allows for efficient communication of disease status between clinicians. Additionally, accurate staging can assist in formulating a prognosis for recovery, as discussed later in this section [17, 56, 62, 63, 69].

Treatment of tick paralysis requires removal of the offending tick (as described above), supportive care of the patient, and, in some patients, administration of tick antiserum (TAS). There are no clear-cut guidelines for when TAS is indicated in cases of tick paralysis. In one nationwide retrospective study, TAS was used in less than 2% of all cases [56]. This study did not report the tick clinical score of these cases and it is unknown if the low rates of TAS administration were due to a predominance of mild cases or other reasons such as financial constraints. This is in stark contrast to a recent retrospective cohort study of 2077 cases of tick paralysis over an eight-year period. In this study, TAS was administered in 95% of the 1742 feline cases where 5-day mortality was known [63]. This study also reports a 4-fold reduction in risk of death when TAS was administered as a part of comprehensive therapy. The authors use the very loose guideline that TAS administration should be discussed with the owner and considered in all tick paralysis patients regardless of stage. However, risk of TAS for some patients remains high enough that these risks may outweigh the benefits in certain patients (e.g., a cat with mild ataxia or previous history of TAS exposure).

The risks of TAS administration include anaphylactic reactions and the Bezold-Jarisch (BJ) reflex [56, 70]. The BJ reflex is a vagally medicated response secondary to direct chemical stimulation of cardiac receptors. Because of this response, it has been advocated to premedicate with atropine prior to administration; however, this recommendation has changed over time and it is common for practitioners to use no premedication prior to administration of TAS [56, 63, 70]. Adverse reactions to TAS have been reported in as many as 9% of cats and 3% of dogs [63, 70]. Anaphylaxis is a serious complication of TAS administration, and for this reason it is recommended that TAS be administered slowly over the first 15 minutes and the patient monitored closely.

Survival rates for most cases of tick paralysis are quite high. Although occasional cases progress to severe disease (Stages 3 and 4, C and D) the majority of cases remain clinically stable or respond quickly to TAS and complete resolution of disease is seen over a period of days [17, 56, 62]. Overall survival rates of upwards of 95% are reported in the literature [56]. However, for patients requiring mechanical ventilation or those experiencing severe complications such as aspiration pneumonia, the prognosis for survival is much more guarded [63, 69].

Due to the varying severity of tick paralysis cases, there is no blanket dose rate of TAS that can be used for paralysis cases. The manufacturer (AVSL) recommends administering TAS at a rate of 1 ml/kg in stage 1A-2B cases of paralysis; however doses of up to 4.0ml/kg may be required. TAS should be allowed to warm to room temperature and then diluted 1:1 with normal saline and administered slowly (over 15-20 minutes) via intravenous route in dogs. In cats, the diluted TAS should be administered more slowly, with a small amount given intravenously over the first 15 minutes while the patient is closely observed for evidence of an adverse reaction. The remainder of the dose can then be administered over 30-60 minutes.

## 5. Snake Envenomation

There are several species of venomous snakes in Australia with venom capable of producing neurologic signs consistent with a lower motor neuron blockade. Nationwide, the most common species responsible for snake bites in small animals are the eastern brown snake (*Pseudonaja textilis*), western brown snake (*Pseudonaja nuchalis*), tiger snakes (*Notechis scutatus*), and red-bellied black snakes (*Pseudechis porphyriacus*) [71–76]. Of these, venoms of the tiger and brown snakes are most likely to produce neurologic signs [8, 72–75, 77–79]. Anecdotally, envenomation from the whipsnake (*Demansia *spp.) is also associated with neurologic signs in dogs, although there are no case series reported in the literature. The most commonly encountered species of snake envenomation will depend largely on the geographical location of the patient at the time of the bite. In one 2005 survey of snake bites of animals treated by veterinarians in New South Wales it was reported that over 40% of snake bites were due to brown snake [72]. An older survey of snake bites in Australia reported over 76% of snake bites treated nationwide were the result of brown snake envenomation [78].

The venom of each of these snakes is a heterogenous mix of toxic compounds and therefore envenomations are infrequently associated with only a single clinical sign [8]. Depending on the dose of venom delivered to the patient, the species of snake, and age of the snake, dogs and cats may present with only weakness and ataxia consistent with lower motor neuron blockade. Neurologic signs are due to both pre- and postsynaptic neurotoxins, inhibiting acetylcholine receptor activity and/or release of acetylcholine vesicles from the nerve terminus [75, 80].

Patients presenting with bites from brown snakes frequently have both neurologic and coagulopathic signs [8, 75]. The venom of juvenile brown snakes is almost exclusively neurotoxic, whereas in adult snakes the venom is both neurotoxic and coagulopathic [81]. Tiger snake bites are frequently associated with neurologic (LMN) signs [71–74, 77–79, 82]. Although black snake venom can produce neurologic signs, it is much more likely to produce myolytic and coagulopathic signs in addition to any neurologic signs in a dog [8, 76, 80, 83]. The presence of a neuropathy with a concurrent coagulopathy or myopathy is most consistent with snake envenomation. Confirmation of snake envenomation can be made with a Snake Venom Detection Kit (SVDK) (CSL Limited, Victoria Park, Australia) using a swab of the bite site, serum/plasma, heparinised whole blood, or urine as the sample. A study in cats showed that plasma is the most reliable sample for detecting envenomation with a SVDK if the bite occurred within the last 8 hours, or urine if the bite occurred greater than 8 hours ago [84]. This is due to a delay of up to eight hours for the toxin to be filtered into the urine by the kidneys. In this same study, venom could be detected in a plasma sample within the first seventeen hours, but venom in the urine was detected up to 48 hours after bite.

There are no veterinary studies investigating the variation of clinical signs associated with various species of snakes within a genus or in various geographic regions. Even among members of the same genus, there can be considerable intra- and interindividual variation in venom composition [5, 85]. In one case series study in human brown snake envenomations across Australia there was no difference in clinical signs, severity of disease, or deaths when cohorts of cases from various regions were compared [86]. Anecdotal reports by veterinary emergency and critical care specialists familiar with brown snake envenomation from both eastern and western brown snakes report that cases associated with eastern brown snake seem to be more likely to have a slightly delayed presentation of neurologic signs (up to 36 hours) as compared to western brown snake bites that appear to present much more rapidly with neurologic signs. The delayed presentation complicates diagnosis as the SDVK may be negative when assessed more than 48 hours after the bite.

As with other causes of LMND, treatment will largely depend on the severity of LMN signs and may include ventilator support, nutritional support, and nursing care while the clinical signs improve. Unless the offending snake was seen and positively identified, it is always preferable to obtain a definitive identification via the SVDK so that antivenin therapy can be as specific as possible.

Using monovalent antivenin is preferred as it is more efficacious and cost effective and has less risk of adverse reactions due to the smaller volume required for treatment as compared to polyvalent. The commercially available antivenin for veterinary use is formulated from hyperimmune equine serum, with each vial containing enough antibody to neutralise the average amount of venom milked from a snake of that species (package insert, Brown Snake Antivenom, AVSL, Lismore, Australia). The amount required, however, will depend on the amount of venom received by the patient [72]. The SVDK provides genus-specific identification of toxin, thereby allowing the practitioner to select the most appropriate monovalent antivenin.

The method of administration of antivenin may depend slightly on manufacturer instructions. Generally room temperature antivenin is diluted with a balanced electrolyte solution (e.g., Hartmann's) at a dilution of 1:10 and then given slowly via intravenous route over 15-30 minutes [72]. The major risk following antivenin administration is anaphylaxis and patients should always be carefully monitored during administration. Those patients who have already received antivenin are at greater risk of an adverse reaction. A delayed, type-III hypersensitivity has been recorded in humans, but has not been recorded in dogs [72]. Premedication with atropine, epinephrine, or a corticosteroid is not recommended.

Prognosis for survival of snake bite envenomation in dogs and cats is good but depends on severity of clinical signs, accurate identification of the snake involved, and treatment with appropriate antivenins. Seventy-five percent of dogs and 91% of cats treated with antivenin survived as compared to only 66% of cats and 31% of dogs that did not receive antivenin in one retrospective study [78]. Another survey suggested that overall survival of snake bites in dogs and cats treated in New South Wales veterinary hospitals was seen in approximately 63, 70, and 84% of cases of tiger, brown, and red belly black snake, respectively [72].

## 6. Tetrodotoxin

An infrequently encountered but important cause of LMND in Australian dogs is ingestion of marine animals, particularly puffer fish or blue-ringed octopus, containing tetrodotoxin. Tetrodotoxin is not exclusive to puffer fish or blue-ring octopus, as the toxin has been identified in some terrestrial frogs and newts as well as several marine sea stars and sea slugs [87–89]. Interestingly, tetrodotoxin is not a direct product of the organism, but rather a product of commensal marine bacteria within and/or ingested by the animal (e.g., puffer fish) [90]. The toxin is not evenly distributed throughout the animal, with high concentrations to be found in the skin and viscera of puffer fish [87]. Dogs may encounter whole fish or parts of fish left on beaches by fishermen as pufferfish are a regular by-catch for sports fishermen in Australia.

Tetrodotoxin is a potent neurotoxin that blocks fast sodium channels both within the nerve axon and on the myocyte. Onset of clinical signs is fairly rapid. In people, clinical signs manifest as numbness in the limbs followed by an ascending LMN paresis and paralysis. Large doses will manifest as seizures, respiratory paralysis, coma, and death [87]. Clinical signs in dogs are believed to include vomiting, ataxia, lethargy, cardiac arrhythmias, respiratory paralysis, and death [88].

There is no antitoxin for tetrodotoxin and treatment is supportive. Diagnosis of tetrodotoxin poisoning should be made based on clinical presentation combined with a recent history of the pet visiting a beach or marina where discarded fish may have been found.

## 7. Miscellaneous Causes

The complete list of diseases, drugs, and toxins associated with neuromuscular blockade or neuromuscular weakness is outside the scope of this review. A brief list of less common causes of significant LMN disease in dogs and cats includes botulism, aminoglycoside antibiotics, hypothyroidism, hyperadrenocorticism, paraneoplastic polyneuritis, and vincristine/vinblastine neuropathy [11, 91–93].

LMN signs associated with botulinum toxin have been reported in dogs but appear to be uncommon to rare [94]. Dogs may develop intoxication after ingestion of a carcass or similar spoiled product [95]. Signs of botulism can be delayed up to 4 days after ingestion of the toxin but typically begin as an ascending flaccid paralysis beginning in the pelvic limbs [95–97]. Similar to polyradiculoneuritis, affected dogs frequently retain the ability to wag their tail [94]. Treatment is supportive and recovery is usually complete within one to two weeks [94, 95].

Neuromuscular blockade can be seen with several commonly used medications to include aminoglycoside antibiotics and tetracycline antibiotics [93]. Lasalocid toxicity from accidental ingestion or contaminated food has been associated with weakness and LMN signs in dogs [98–100]. Myasthenia gravis has been documented secondary to methimazole therapy in cats within the first several weeks of initiating therapy [101].

Muscle weakness can also be seen in primary myopathies. Therefore, other differentials for patients presenting with presumed LMN or motor unit dysfunction may also include inflammatory causes of muscle weakness such as protozoal disease, immune-mediated myopathies, or paraneoplastic syndromes. A complete discussion of diseases producing a myopathy is beyond the scope of this review.

## 8. Conclusion

Lower motor neuron (motor unit) disease is a frequently encountered complaint in Australian dogs and cats. The frequency of patients presenting with LMN signs is largely due to the unique, native fauna of Australia. Although tick paralysis, snake envenomation, and marine animal intoxications are not exclusive to Australia, they are an important and not-uncommon group of intoxications seen by small animal practitioners in Australia. The ability to both rapidly identify LMN disease signs and identify the most likely diseases or intoxications associated with LMN disease in Australian dogs and cats are key to successful treatment and positive outcomes.

## Figures and Tables

**Table 1:** Summary of abnormal findings on a clinical neurological exam and whether these findings are consistent with lower motor neuron disease.

**Neurological Exam Abnormality**	**Typical of LMND**
**Seizures**	NO
**Altered mentation**	
**Pacing**	
**Head pressing**	
**Head tilt**	
**Head turn**	

**Gait abnormalities**	
** Short gait, stilted gait, sits frequently **	YES
** Ataxia**	
** Normal muscle tone, abnormal movement**	NO
** Hypermetria**	NO
** Lameness**	NO
** Tires easily/weakness after exercise**	YES

**Proprioception/Postural reactions are** ABNORMAL or ABSENT	NO
** ** **∗** **∗** **Only evaluate when patient is PROPERLY SUPPORTED when reactions are tested.** **∗** **∗**	

**Decreased muscle tone and/or muscle *atrophy***	YES

**Spinal Reflexes**	
** patellar, triceps, perineal, sciatic DIMINISHED or EXHAUSTABLE with repetition**	YES, although perineal reflexes and motor function to tail may be preserved
** Reflexes clonic or exaggerated**	NO

**Nociception diminished or absent**	NO

**Dysphonia**	YES
**Dysphagia**	

**Spinal pain**	NO (rare with acute PRN)

**Cranial nerves**	
** Bilateral abnormalities in PLR, facial nerve weakness, diminished swallow or gag**	Not typical of ALL LMND, but common with tick paralysis
** UNILATERAL abnormalities?**	NO

**Megaoesophagus**	Not typical of all LMND, but frequently seen with MG and tick paralysis

**Table 2:** Clinical scoring system used to standardise the clinical severity of tick paralysis in dogs and cats. Adapted from *Atwell et al., 2001*.

**Neuromuscular Score**		**Respiratory Signs**	
**1**	Normal or mild weakness and incoordination	**A**	Normal

**2**	Ambulatory but with obvious weakness	**B**	Increased respiratory rate, but normal effort

**3**	Unable to stand, but can right self	**C**	Restrictive breathing pattern, gagging, retching

**4**	Unable to right self, moribund	**D**	Expiratory grunt, dyspnoea, cyanosis

## References

[1] Cuddon P. A. (1998). Electrophysiologic Assessment of Acute Polyradiculoneuropathy in Dogs: Comparison with Guillain-Barré Syndrome in People. *Journal of Veterinary Internal Medicine*.

[2] Olby N. (2004). Motor neuron disease: Inherited and acquired. *Veterinary Clinics of North America - Small Animal Practice*.

[3] Shelton G. D. (2016). Myasthenia gravis and congenital myasthenic syndromes in dogs and cats: A history and mini-review. *Neuromuscular Disorders*.

[4] Barker S. C., Walker A. R. (2014). Ticks of Australia. The species that infest domestic animals and humans. *Zootaxa*.

[5] Birrell G. W., Earl S., Masci P. P. (2006). Molecular diversity in venom from the Australian brown snake, Pseudonaja textilis. *Molecular & Cellular Proteomics*.

[6] Brazier I., Kelman M., Ward M. P. (2014). The association between landscape and climate and reported tick paralysis cases in dogs and cats in Australia. *Veterinary Parasitology*.

[7] Greay T. L., Oskam C. L., Gofton A. W., Rees R. L., Ryan U. M., Irwin P. J. (2016). A survey of ticks (Acari: Ixodidae) of companion animals in Australia. *Parasites & Vectors*.

[8] Heller J., Mellor D. J., Hodgson J. L., Reid S. W. J., Hodgson D. R., Bosward K. L. (2007). Elapid snake envenomation in dogs in New South Wales: a review. *Australian Veterinary Journal*.

[9] Rika-Heke T., Kelman M., Ward M. P. (2015). The relationship between the Southern Oscillation Index, rainfall and the occurrence of canine tick paralysis, feline tick paralysis and canine parvovirus in Australia. *The Veterinary Journal*.

[10] Hegyi B., Bárándi L., Komáromi I. (2012). Tetrodotoxin blocks L-type Ca 2+ channels in canine ventricular cardiomyocytes. *Pflügers Archiv - European Journal of Physiology*.

[11] Añor S. (2014). Acute lower motor neuron tetraparesis. *Veterinary Clinics of North America - Small Animal Practice*.

[12] Chand K. K., Lee K. M., Lavidis N. A. (2016). Tick holocyclotoxins trigger host paralysis by presynaptic inhibition. *Scientific Reports*.

[13] Flagstad A. (1991). Myasthenia in dogs. *Tijdschrift voor diergeneeskunde*.

[14] Holland C. T. (2008). Asymmetrical focal neurological deficits in dogs and cats with naturally occurring tick paralysis (Ixodes holocyclus): 27 cases (1999-2006). *Australian Veterinary Journal*.

[15] Padula A. M., Gopalakrishnakone P., Faiz S. M. A., Gnanathasan C. A. (2016). Tick Paralysis of Animals in Australia. *Clinical Toxinology: Clinical Toxinology*.

[16] Webb A. A., Taylor S. M., McPhee L. (1997). Focal myasthenia gravis in a dog. *Canadian Veterinary Journal*.

[17] Atwell R. B., Campbell F. E., Evans E. A. (2001). Prospective survey of tick paralysis in dogs. *Australian Veterinary Journal*.

[18] Dewey C. W., Bailey C. S., Shelton G. D., Kass P. H., Cardinet 3rd. G. H. (1997). Clinical forms of acquired myasthenia gravis in dogs: 25 cases (1988-1995).. *Journal of veterinary internal medicine / American College of Veterinary Internal Medicine*.

[19] Pecina C. A. (2012). Tick paralysis. *Seminars in Neurology*.

[20] Shelton G. D. (2002). Myasthenia gravis and disorders of neuromuscular transmission. *Veterinary Clinics of North America - Small Animal Practice*.

[21] Shelton G. D., Willard M. D., III G. H. C., Lindstrom J. (1990). Acquired Myasthenia Gravis: Selective Involvement of Esophageal, Pharyngeal, and Facial Muscles. *Journal of Veterinary Internal Medicine*.

[22] Hirschvogel K., Jurina K., Steinberg T. A. (2012). Clinical course of acute canine polyradiculoneuritis following treatment with human IV immunoglobulin. *Journal of the American Animal Hospital Association*.

[23] Rutter C. R., Rozanski E. A., Sharp C. R., Powell L. L., Kent M. (2011). Outcome and medical management in dogs with lower motor neuron disease undergoing mechanical ventilation: 14 cases (2003-2009). *Journal of Veterinary Emergency and Critical Care*.

[24] Cummings J. F., Haas D. C. (1967). Coonhound paralysis. An acute idiopathic polyradiculoneuritis in dogs resembling the Landry-Guillain-Barré syndrome. *Journal of the Neurological Sciences*.

[25] Holmes D. F., Schultz R. D., Cummings J. F., Delahunta A. (1979). Experimental coonhound paralysis: Animal model of guillain-barre syndrome. *Neurology*.

[26] Kingma G., Catcott E. (1954). A paralytic syndrome in coonhounds. *Veterinary Clinics of North America*.

[27] Gehring R., Eggars B. (2001). Suspected post-vaccinal acute polyradiculoneuritis in a puppy. *Journal of the South African Veterinary Association*.

[28] Gutierrez-Quintana R., Cuesta-Garcia N., Wessmann A., Johnston P., Penderis J. (2015). Acute motor and sensory polyganglioradiculoneuritis in a cat: clinical and histopathological findings. *Journal of Feline Medicine and Surgery*.

[29] Holt N., Murray M., Cuddon P. A., Lappin M. R. (2011). Seroprevalence of Various Infectious Agents in Dogs with Suspected Acute Canine Polyradiculoneuritis. *Journal of Veterinary Internal Medicine*.

[30] Laws E. J., Harcourt-Brown T. R., Granger N., Rose J. H. (2017). An exploratory study into factors influencing development of acute canine polyradiculoneuritis in the UK. *Journal of Small Animal Practice*.

[31] Rupp A., Galban-Horcajo F., Bianchi E. (2013). Anti-GM2 ganglioside antibodies are a biomarker for acute canine polyradiculoneuritis. *Journal of the Peripheral Nervous System*.

[32] Henke D., Vandevelde M., Oevermann A. (2009). Polyganglioradiculoneuritis in a young cat: Clinical and histopathological findings. *Journal of Small Animal Practice*.

[33] Stanciu G. D., Solcan G. (2016). Acute idiopathic polyradiculoneuritis concurrent with acquired myasthenia gravis in a West Highland white terrier dog. *BMC Veterinary Research*.

[34] Gerritsen R. J., van Nes J. J., van Niel M. H. F., van den Ingh T. S. G. A. M., Wijnberg I. D. (1996). Acute idiopathic polyneuropathy in nine cats. *Veterinary Quarterly*.

[35] Hughes R. A., Swan A. V., van Koningsveld R., van Doorn P. A. (2006). Corticosteroids for Guillain-Barré syndrome.. *Cochrane Database of Systematic Reviews (Online)*.

[36] Hague D. W., Humphries H. D., Mitchell M. A., Shelton G. D. (2015). Risk Factors and Outcomes in Cats with Acquired Myasthenia Gravis (2001-2012). *Journal of Veterinary Internal Medicine*.

[37] Dewey C. W., Bailey C. S., Shelton G. D., Kass P. H., III G. H. (1997). Clinical Forms of Acquired Myasthenia Gravis in Dogs: 25 Cases (1988-1995). *Journal of Veterinary Internal Medicine*.

[38] Dickinson P. J., Sturges B. K., Shelton G. D., LeCouteur R. A. (2005). Congenital Myasthenia Gravis in Smooth-Haired Miniature Dachshund Dogs. *Journal of Veterinary Internal Medicine*.

[39] Shelton G. D., Mindy H., Kass P. H. (2000). Risk factors for acquired myasthenia gravis in cats: 105 cases (1986-1998). *Journal of the American Veterinary Medical Association*.

[40] Hill P. B., Brain P., Collins D., Fearnside S., Olivry T. (2013). Putative paraneoplastic pemphigus and myasthenia gravis in a cat with a lymphocytic thymoma. *Veterinary Dermatology*.

[41] Krotje L. J., Fix A. S., Potthoff A. D. (1990). Acquired myasthenia gravis and cholangiocellular carcinoma in a dog. *Journal of the American Veterinary Medical Association*.

[42] Moffet A. C. (2007). Metastatic thymoma and acquired generalized myasthenia gravis in a beagle. *Canadian Veterinary Journal*.

[43] Stepaniuk K., Legendre L., Watson S. (2011). Acquired myasthenia gravis associated with oral sarcoma in a dog. *Journal of Veterinary Dentistry*.

[44] Shelton G. D. (1999). Acquired myasthenia gravis: What we have learned from experimental and spontaneous animal models. *Veterinary Immunology and Immunopathology*.

[45] Shelton G. D. (1998). Myasthenia gravis: Lessons from the past 10 years. *Journal of Small Animal Practice*.

[46] Dewey C. W., Cerda-Gonzalez S., Fletcher D. J. (2010). Mycophenolate mofetil treatment in dogs with serologically diagnosed acquired myasthenia gravis: 27 cases (1999-2008. *Journal of the American Veterinary Medical Association*.

[47] Abelson A. L., Shelton G. D., Whelan M. F., Cornejo L., Shaw S., O'Toole T. E. (2009). Use of mycophenolate mofetil as a rescue agent in the treatment of severe generalized myasthenia gravis in three dogs. *Journal of Veterinary Emergency and Critical Care*.

[48] Dewey C. W., Coates J. R., Ducoté J. M., Meeks J. C., Fradkin J. M. (1999). Azathioprine therapy for acquired myasthenia gravis in five dogs. *Journal of the American Animal Hospital Association*.

[49] Bartges J. W., Klausner J. S., Bostwick E. F., Hakala J. E., Lennon V. A. (1990). Clinical remission following plasmapheresis and corticosteroid treatment in a dog with acquired myasthenia gravis.. *Journal of the American Veterinary Medical Association*.

[50] Guptill J. T., Juel V. C., Massey J. M. (2016). Effect of therapeutic plasma exchange on immunoglobulins in myasthenia gravis. *Autoimmunity*.

[51] Lizarraga A. A., Lizarraga K. J., Benatar M. (2016). Getting Rid of Weakness in the ICU: An Updated Approach to the Acute Management of Myasthenia Gravis and Guillain-Barré Syndrome. *Seminars in Neurology*.

[52] Ortiz-Salas P., Velez-Van-Meerbeke A., Galvis-Gomez C. A., Rodriguez J. H. (2016). Human immunoglobulin versus plasmapheresis in guillain-barre syndrome and myasthenia gravis: A meta-analysis. *Journal of Clinical Neuromuscular Disease*.

[53] Shelton G. D., Lindstrom J. M. (2001). Spontaneous remission in canine myasthenia gravis: Implications for assessing human MG therapies. *Neurology*.

[54] Diaz J. H. (2010). Tick paralysis in the United States. *The Journal of the Louisiana State Medical Society*.

[55] Diaz J. H. (2015). A Comparative Meta-Analysis of Tick Paralysis in the United States and Australia. *Clinical Toxicology*.

[56] Eppleston K. R., Kelman M., Ward M. P. (2013). Distribution, seasonality and risk factors for tick paralysis in Australian dogs and cats. *Veterinary Parasitology*.

[57] Barker S. C., Walker A. R., Campelo D. (2014). A list of the 70 species of Australian ticks; diagnostic guides to and species accounts of Ixodes holocyclus (paralysis tick), Ixodes cornuatus (southern paralysis tick) and Rhipicephalus australis (Australian cattle tick); and consideration of the place of Australia in the evolution of ticks with comments on four controversial ideas. *International Journal for Parasitology*.

[58] Hall-Mendelin S., Craig S. B., Hall R. A. (2011). Tick paralysis in Australia caused by Ixodes holocyclus Neumann. *Annals of Tropical Medicine and Parasitology*.

[59] Ilkiw J. E., Turner D. M., Howlett C. R. (1987). Infestation in the dog by the paralysis tick Ixodes holocyclus. 1. Clinical and histological findings.. *Australian Veterinary Journal*.

[60] Guernier V., Milinovich G. J., Santos M. A. B., Haworth M., Coleman G., Soares Magalhaes R. J. (2016). Use of big data in the surveillance of veterinary diseases: Early detection of tick paralysis in companion animals. *Parasites & Vectors*.

[61] Jackson J., Beveridge I., Chilton N. B., Andrews R. H. (2007). Distributions of the paralysis ticks Ixodes cornuatus and Ixodes holocyclus in south-eastern Australia. *Australian Veterinary Journal*.

[62] Schull D. N., Litster A. L., Atwell R. B. (2007). Tick toxicity in cats caused by Ixodes species in Australia: a review of published literature. *Journal of Feline Medicine and Surgery*.

[63] Leister E., Morton J., Atwell R., Webster R. (2017). Clinical presentations, treatments and risk factors for mortality in cats with tick paralysis caused by. *Journal of Feline Medicine and Surgery*.

[64] de Burgh S., Hunter K., Jackson C., Chambers M., Klupiec C., Smith V. (2017). Repellency Effect of an Imidacloprid / Flumethrin (Seresto®) Controlled Release Polymer Matrix Collar against the Australian Paralysis Tick (Ixodes holocyclus) in Dogs. *Parasitology Research*.

[65] Packianathan R., Hodge A., Bruellke N., Davis K., Maeder S. (2017). Comparative speed of kill of sarolaner (Simparica®) and afoxolaner (NexGard®) against induced infestations of Ixodes holocyclus on dogs. *Parasites & Vectors*.

[66] Smith W. M., Ahlstrom L. A., Rees R. (2013). Long-term efficacy of an imidacloprid 10 % / flumethrin 4.5 % polymer matrix collar (seresto®, bayer) against the australian paralysis tick (ixodes holocyclus) in dogs. *Parasitology Research*.

[67] Webster M. C., Fisara P., Sargent R. M. (2011). Long-term efficacy of a deltamethrin-impregnated collar for the control of the Australian paralysis tick, Ixodes holocyclus, on dogs. *Australian Veterinary Journal*.

[68] Rodd A., Leister E. (2017). Time series analysis of tick paralysis cases in south-eastern queensland over a 9-year period. Australia and New Zealand College of Veterinary Scientists (ANZCVS) Science Week. *Gold Coast*.

[69] Webster R., Mills P., Morton J. (2013). Indications, durations and outcomes of mechanical ventilation in dogs and cats with tick paralysis caused by Ixodes holocyclus: 61 cases (2008-2011). *Australian Veterinary Journal*.

[70] Atwell R. B., Campbell F. E. (2001). Reactions to tick antitoxin serum and the role of atropine in treatment of dogs and cats with tick paralysis caused by Ixodes holocyclus: A pilot survey. *Australian Veterinary Journal*.

[71] BARR S. C. (1984). Clinical features therapy and epidemiology of tiger snake bite in dogs and cats. *Australian Veterinary Journal*.

[72] Heller J., Bosward K. L., Hodgson J. L. (2005). Snake envenomation in dogs in New South Wales. *Australian Veterinary Journal*.

[73] Hill F. W. G. (1979). Snake bite in dogs. *Australian Veterinary Journal*.

[74] Hill F. W. G., Campbell T. (1978). SNAKE BITE IN CATS. *Australian Veterinary Journal*.

[75] Padula A. M., Leister E. (2017). Eastern brown snake (Pseudonaja textilis) envenomation in dogs and cats: Clinical signs, coagulation changes, brown snake venom antigen levels and treatment with a novel caprylic acid fractionated bivalent whole IgG equine antivenom. *Toxicon*.

[76] Padula A. M., Winkel K. D. (2016). Red-bellied black snake (Pseudechis porphyriacus) envenomation in the dog: Diagnosis and treatment of nine cases. *Toxicon*.

[77] Hopper K., Beck C., Slocombe R. F. (2001). Megaoesophagus in adult dogs secondary to Australian tiger snake envenomation. *Australian Veterinary Journal*.

[78] Mirtschin P. J., Masci P., Paton D. C., Kuchel T. (1998). Snake bites recorded by veterinary practices in Australia. *Australian Veterinary Journal*.

[79] Padula A. M., Winkel K. D. (2016). Fatal presumed tiger snake (Notechis scutatus) envenomation in a cat with measurement of venom and antivenom concentration. *Toxicon*.

[80] Ramasamy S., Fry B. G., Hodgson W. C. (2005). Neurotoxic effects of venoms from seven species of Australasian black snakes (Pseudechis): Efficacy of black and tiger snake antivenoms. *Clinical and Experimental Pharmacology and Physiology*.

[81] Cipriani V., Debono J., Goldenberg J. (2017). Correlation between ontogenetic dietary shifts and venom variation in Australian brown snakes (Pseudonaja). *Comparative Biochemistry and Physiology Part - C: Toxicology and Pharmacology*.

[82] Jacoby-Alner T. E., Stephens N., Davern K. M., Balmer L., Brown S. G. A., Swindells K. (2011). Histopathological analysis and in situ localisation of Australian tiger snake venom in two clinically envenomed domestic animals. *Toxicon*.

[83] Padula A. M., Leister E. M. (2017). Severe neurotoxicity requiring mechanical ventilation in a dog envenomed by a red-bellied black snake (Pseudechis porphyriacus) and successful treatment with an experimental bivalent whole equine IgG antivenom. *Toxicon*.

[84] Moisidis A. V., James T., Smith H. V., Cox J. C. (1996). Snake envenomation in cats and its detection by rapid immunoassay. *Australian Veterinary Journal*.

[85] Reeks T., Lavergne V., Sunagar K. (2016). Deep venomics of the Pseudonaja genus reveals inter- and intra-specific variation. *Journal of Proteomics*.

[86] Allen G. E., Brown S. G. A., Buckley N. A. (2012). Clinical Effects and Antivenom Dosing in Brown Snake (Pseudonaja spp.) Envenoming - Australian Snakebite Project (ASP-14). *PLoS ONE*.

[87] Noguchi Tamao, Onuki Kazue, Arakawa Osamu (2011). Tetrodotoxin Poisoning Due to Pufferfish and Gastropods, and Their Intoxication Mechanism. *ISRN Toxicology*.

[88] McNabb P., Selwood A. I., Munday R. (2010). Detection of tetrodotoxin from the grey side-gilled sea slug - Pleurobranchaea maculata, and associated dog neurotoxicosis on beaches adjacent to the Hauraki Gulf, Auckland, New Zealand. *Toxicon*.

[89] Lorentz M. N., Stokes A. N., Rößler D. C., Lötters S. (2016). Tetrodotoxin. *Current Biology*.

[90] Pratheepa V., Vasconcelos V. (2013). Microbial diversity associated with tetrodotoxin production in marine organisms. *Environmental Toxicology and Pharmacology*.

[91] Cuddon P. A. (2002). Acquired canine peripheral neuropathies. *Veterinary Clinics of North America - Small Animal Practice*.

[92] Hamilton T. A., Cook. J. R., Braund K. G. (1991). Vincristine-induced peripheral neuropathy in a dog. *Journal of the American Veterinary Medical Association*.

[93] Howard J. F., Sanders D. B. (2008). Chapter 12 Neurotoxicology of neuromuscular transmission. *Handbook of Clinical Neurology*.

[94] Sykes J. E., Saunders W. B. (2014). Chapter 54 - Tetanus and Botulism. *Canine and Feline Infectious Diseases*.

[95] Farrow B. R. H., Murrell W. G., Revington M. L., Stewart B. J., Zuber R. M. (1983). Type C botulism in young dogs. *Australian Veterinary Journal*.

[96] Bruchim Y., Steinman A., Markovitz M., Baneth G., Elad D., Shpigel N. Y. (2006). Toxicological, bacteriological and serological diagnosis of botulism in a dog. *Veterinary Record*.

[97] Elad D., Yas-Natan E., Aroch I. (2004). Natural Clostridium botulinum type C toxicosis in a group of cats. *Journal of Clinical Microbiology*.

[98] Espino L., Suarez M. L., Mino N. (2003). Suspected lasalocid poisoning in three dogs. *Veterinary and human toxicology*.

[99] Safran N., Aizenberg I., Bark H. (1993). Paralytic syndrome attributed to lasalocid residues in a commercial ration fed to dogs.. *Journal of the American Veterinary Medical Association*.

[100] Segev G., Baneth G., Levitin B., Shlosberg A., Aroch I. (2004). Accidental poisoning of 17 dogs with lasalocid. *Veterinary Record*.

[101] Bell E. T., Mansfield C. S., James F. E. (2012). Immune-mediated myasthenia gravis in a methimazole-treated cat. *Journal of Small Animal Practice*.

